# Case report: a fatal case of disseminated adenovirus infection in a non-transplant adult haematology patient

**DOI:** 10.1186/s12879-018-2962-7

**Published:** 2018-01-27

**Authors:** Michael Joffe, Simon D. Wagner, Julian W. Tang

**Affiliations:** 1 Department of Haematology, Leicester Royal Infirmary, University Hospitals of Leicester NHS Trust, Leicester, UK; 2 Clinical Microbiology, Leicester Royal Infirmary, University Hospitals of Leicester NHS Trust, Level 5 Sandringham Building, Leicester Royal Infirmary, Infirmary Square, Leicester, LE1 5WW UK; 3 Department of Infection, Immunity and Inflammation, University of Leicester, Leicester, UK

**Keywords:** Case report, Adenovirus, Respiratory, Disseminated infection, Fatal, PCR, Earlier, Treatment

## Abstract

**Background:**

We report a fatal case of disseminated adenovirus infection in a non-transplant haematology adult patient with chronic lymphocytic leukaemia who had completed combination chemoimmunotherapy a few months before developing respiratory symptoms. In such non-transplant patients, monitoring for adenovirus in the blood is not routine. However, with adenoviruses, when there is a more peripheral (i.e. non-blood) site of infection such as the chest, serial adenovirus monitoring in blood for the duration of that illness may be warranted.

**Case presentation:**

This case started with an initial bacterial chest infection that responded to treatment, followed by an adenovirus pneumonitis that disseminated to his blood a week later with levels of up to 92 million adenovirus DNA copies/ml. Despite prompt treatment with cidofovir, his respiratory function continued to deteriorate over the next two weeks and he was moved to intensive care. Intravenous immunoglobulin and ribavirin were subsequently added to his treatment. However, he died soon after this with a final adenovirus load of 20 million copies/ml in his blood.

**Conclusions:**

We recommend that even in non-transplant haematology patients, where such patients present with an acute respiratory adenovirus infection, teams should consider checking the blood for adenovirus to check for signs of disseminated infection. The earlier this can be tested, the earlier treatment can be initiated (if adenovirus positive), which may produce more successful clinical outcomes.

## Background

Human adenoviruses are non-enveloped, double-stranded DNA viruses. They exist as seven species (A-G) with greater than 50 types identified so far that can affect different organs and cause a wide spectrum of disease in both immunocompetent [1–4] and immunocompromised [1, 5–8] individuals, including pneumonitis, hepatitis, gastroenteritis and conjunctivitis [1]. Although adenoviruses are important respiratory pathogens in haematology patients, adenovirus surveillance in blood is not normally performed in non-transplant adult patients. We report here a case of adenovirus pneumonitis which led to a fatal disseminated adenovirus infection in an adult patient with chronic lymphocytic leukaemia (CLL) on chemotherapy.

## Case report

The patient was a 58-year old man with CLL, who presented with persistent cough and coryzal symptoms in early 2015. He had a past medical history of epilepsy and rheumatic fever. He had never smoked. CLL was first diagnosed in late 2013. The leukaemia progressed and he underwent six cycles of chemotherapy with the FCR combination (fludarabine 24 mg/m^2^, cyclophosphamide 150 mg/m^2^ and rituximab 375 mg/m^2^) over the next 5 months completing the treatment in June 2014. Bone marrow and clinical findings showed a complete response to treatment.

After his chemoimmunotherapy he continued on acyclovir (400 mg BD) and co-trimoxazole (960 mg BD, 3 times a week) prophylaxis, both of which he was still taking at the time of presentation. This was in response to a T-cell lymphopenia that persisted up to his hospital admission in July 2015 (CD4^+^ T helper cells 0.24 × 10 [9]/L).

In April 2015 he presented with a 4-week history of a non-productive cough and rhinorrhoea. There were no clinical or laboratory features of CLL at that time but serum immunoglobulins were suppressed (IgG 3.2 g/L (normal range 6–16 g/L) and CD4^+^ T-cells were low at 0.24 × 10 [9]/L (normal range 0.49 to 1.69 × 10 [9]/L). Although there were transient improvements, productive cough persisted despite multiple courses of antibiotics (azithromycin 500 mg OD, co-amoxiclav 625 mg TDS, doxycycline 100 mg OD) and a course of oral prednisolone (40 mg OD). A CT scan of his sinuses showed right maxillary sinus change consistent with chronic sinusitis, and he was subsequently referred to ear nose and throat specialists for further management. However, the radiological sinus changes were not felt to be significant and no specific treatment was initiated.

The patient’s symptoms worsened and in the June 2015 a CT scan of the chest showed centrilobular nodular changes, right-sided patchy consolidation with surrounding ground glass opacity with halo sign. This raised the possibility of atypical infection including fungal pathogens and the patient was subsequently prescribed voriconazole 200 mg BD. He also underwent a bronchoscopy later the same month, from which a broncho-alveolar lavage (BAL) sample cultured *Haemophilus influenzae,* but was negative for other pathogens including fungi. No viral screen was carried out on this sample. In response to this, he was given a prolonged course of co-amoxiclav (625 mg TDS). He showed subsequent clinical improvement on this, together with the voriconazole.

One month later (July 2015), however, he was readmitted with a marked deterioration in the productive cough and shortness of breath on exertion. Bilateral crepitations were heard on examination, which was consistent with a chest x-ray showing bilateral, patchy consolidation. C-reactive protein was 308 mg/L. He was started on intravenous (IV) tazocin (1.2 g TDS) and clarithromycin (500 mg BD). Although there were signs of improvement over the course of the week his symptoms persisted.

A repeat CT scan on this readmission showed ground glass changes, tree-in-bud appearance, and nodular changes all of which had progressed from the previous imaging. He underwent another bronchoscopy examination, and was started on a treatment dose of co-trimoxazole (120 mg/kg, daily in divided doses) and caspofungin (50 mg OD). In addition his serum immunoglobulins, which had remained low since his chemotherapy demonstrated pan-hypogammaglobulinaemia, and intravenous immunoglobulin (IVIg) 0.4 g/kg was administered.

This second BAL was positive for adenovirus DNA by PCR testing, using an in-house respiratory multiplex PCR screening assay, as described elsewhere [9]. A beta-D-glucan test on the BAL was also positive at 85 pg/ml, having been negative in the peripheral blood. Fungal, bacterial, pneumocystis and tuberculosis screens were all negative. Despite this, a left mid-zone consolidation persisted on chest imaging. At this point he was diagnosed with adenovirus pneumonitis.

One week later the patient remained symptomatic with persistent fevers, and peripheral blood was sent for adenovirus PCR, with a result of 8.3 million copies/ml. The qualitative and quantitative adenovirus PCR testing on this blood sample were performed at a commercial laboratory (Micropathology Ltd., Coventry, UK). Four days later this adenovirus level had risen to 37.5 million copies/ml (adenovirus type C1, based on viral sequencing and analysis performed by Micropathology Ltd., Coventry, UK). Based on these results IV cidofovir (5 mg/kg weekly) treatment was given.

Four days later the blood adenovirus DNA levels had increased to 92 million copies/ml. Over the course of the following two weeks the patient deteriorated in terms of respiratory function, requiring transfer to intensive care for ventilatory support. His liver function also deteriorated during this time (bilirubin 92 μmol, alkaline phosphatase 676 iu/L. Despite additional measures including further dosing with IVIg (0.4 g/kg), and the addition of ribavirin (IV 33 mg/kg), he died as a result of disseminated adenovirus infection and multi-organ failure. The last adenovirus DNA level, three days before death was 20 million copies/ml.

No post-mortem investigations were performed. Thus, the disseminated adenovirus infection was deemed to be the cause of the patient’s multi-organ failure and death on the basis of the high levels of viremia, which coincided with the patient’s rapid deterioration, as described in other cases [5–8].

## Discussion

There are several well-known risk factors for severe adenovirus infections, including: allogeneic stem cell (or solid-organ) transplantation, particularly with T-cell depletion; treatment with anti-CD52 monoclonal antibody (alemtuzumab or Campath) or anti-thymocyte globulin (ATG); severe immunosuppression used to treat graft-versus-host disease; and any other cause of severe lymphopaenia that reduces the ability of the host’s cell-mediated immunity to defend against adenovirus infection [1].

This patient’s chemotherapy regimen included fludarabine which has severe lymphopaenia as a recognised adverse effect, and which has been present in treatment regimens where various other viral reactivations have occurred, including hepatitis B [10–12], BK virus [13], herpes simplex and Epstein-Barr viruses [14], cytomegalovirus [15], as well as adenovirus [16]. Yet in this case, it was noted that throughout this period during which he acquired and was infected with adenovirus, his total lymphocyte count remained within or even above the normal range of 1–4 × 10 [9]/L, though their specific functionality was not tested.

Although fatal adenovirus infection has been reported in non-transplant paediatric and adult patients on chemotherapy [5–8], there is still no consensus on how to deal with an isolated adenovirus positive result in a peripheral (non-blood) sample type on a routine basis – many such patients also have asymptomatic adenovirus infections. From a virological viewpoint, since all systemic antiviral drugs are only virostatic and not virucidal, earlier adenovirus PCR blood testing allowing earlier treatment to prevent further increases in viral load, may improve clinical outcomes [1]. However, due to the severe nephrotoxic nature of the mainstay treatment, intravenous cidofovir, most transplant teams are reluctant to prescribe this drug empirically, unless a definitive upward trend is seen in the adenovirus blood levels. This is often the cause of delays in commencing therapy with this drug for disseminated adenovirus infection.

## Conclusions

Thus, in such immunocompromised patients where a peripheral site (e.g. a non-blood sample, such as a respiratory or stool sample) has had an adenovirus PCR positive result, we would recommend that serial (once or twice weekly) monitoring for adenovirus PCR testing be performed for the duration of that specific AdV illness, to check for possible disseminated adenovirus infection, earlier.

Such a test in this context is inexpensive and would allow earlier detection of disseminated adenovirus infection. This in turn then allows treatment to be commenced earlier, which could have a significant, positive clinical impact.

Finally in such immunocompromised patients, all pathogens: bacteria, fungi and viruses, should be screened for in the initial investigation of an acute infective episode, to allow prompt intervention as required.

## Abbreviations

ATG: Anti-thymocyte globulin
BAL: Broncho-alveolar lavage
BD: Twice daily
CLL: Chronic lymphocytic leukaemia
CT: Computed tomographic scan
DNA: Deoxyribonucleic acid
FCR: Fludarabine, cyclophosphamide, rituximab chemotherapy regimen
IV: Intravenous
IVIg: Intravenous immunoglobulin
OD: Once daily
PCR: Polymerase chain reaction
TDS: Three times daily
